# Needs and implementation pathways of discharge management in rural areas of Mecklenburg-Western Pomerania: Findings from focus group discussions with multidisciplinary healthcare stakeholders

**DOI:** 10.3205/000350

**Published:** 2025-11-14

**Authors:** Jann Niklas Vogel, Jaqueline Letzin, Hanna Hilgenhof, Ivonne Honekamp, Chiara Kleinschmidt, Anne Petereit, Stefan Schmidt

**Affiliations:** 1 University of Applied Sciences Neubrandenburg, Faculty of Health, Nursing, Administration, Neubrandenburg, Germany; 2 Stralsund University of Applied Sciences, School of Business Studies, Stralsund, Germany

**Keywords:** discharge management, care transition, care coordination, healthcare management, follow-up care

## Abstract

**Background:**

In rural areas, discharge management presents a significant challenge. Despite its relevance, there is limited research on implementation strategies and the preferences of the involved parties. In Mecklenburg-Western Pomerania, four round tables have been established with the goal of promoting sustainable local healthcare. These regional networks involve multidisciplinary stakeholders working together on acute care and follow-up care for patients. This paper examines the implementation strategies of discharge management in rural areas of Mecklenburg-Western Pomerania, analyses associated challenges, and identifies stakeholder preferences.

**Methodology:**

Semi-structured group discussions were conducted at the round tables in Demmin, Pasewalk, and Ueckermünde. The data was analysed using qualitative content analysis, with a focus on structuring the content.

**Results:**

Three group discussions with a total of 30 participants were held. Key challenges in rural areas include securing day care services, the lack of sufficient and locally accessible short-term care, long-term inpatient care, and rehabilitation placements. As a result, patients often remain hospitalized longer than necessary or must transfer to distant follow-up facilities.

**Discussion and conclusion:**

A key aspect is questioning traditional approaches, such as the strict separation of responsibilities, and focusing more on shared accountability. The goal is to create synergies and enable more efficient care delivery.

## Zusammenfassung

**Hintergrund:**

In ländlichen Regionen ist das Entlassmanagement eine Herausforderung. Trotz der thematischen Relevanz liegen nur wenig Forschungsergebnisse zu Umsetzungswegen und Präferenzen der Beteiligten vor. In Mecklenburg-Vorpommern haben sich vier Runde Tische mit dem Ziel gebildet, die lokale Gesundheitsversorgung nachhaltig zu gestalten. In diesen regionalen Netzwerken arbeiten multidisziplinäre Akteur*innen an der Akut- und Nachsorge von Patient*innen zusammen. Der vorliegende Beitrag untersucht Umsetzungswege des Entlassmanagements in ländlichen Räumen von Mecklenburg-Vorpommern, analysiert Herausforderungen und identifiziert Präferenzen von Akteur*innen.

**Methodik:**

Es wurden leitfadenbasierte Gruppendiskussionen an Runden Tischen in Demmin, Pasewalk und Ueckermünde durchgeführt. Die Auswertung erfolgte nach der inhaltlich strukturierenden qualitativen Inhaltsanalyse.

**Ergebnisse:**

Es wurden drei Gruppendiskussionen mit insgesamt 30 Personen durchgeführt. Zentrale Herausforderungen im ländlichen Raum sind die Sicherung der Tagespflege, der Mangel an ausreichender und wohnortnaher Kurzzeitpflege, stationärer Langzeitpflege sowie Reha-Plätzen. Das führt dazu, dass Patient*innen teils länger im Krankenhaus verbleiben als notwendig oder in entfernte Nachsorgeeinrichtungen verlegt werden.

**Diskussion und Schlussfolgerung:**

Ein zentraler Aspekt ist, traditionelle Denkweisen wie die strikte Trennung von Zuständigkeiten zu hinterfragen und gemeinsame Verantwortlichkeiten stärker in den Fokus zu rücken. Ziel ist es, Synergien zu schaffen und eine effizientere Versorgung zu ermöglichen.

## 1 Background

Discharge management (DM) refers to a structured and goal-oriented process facilitating the transition from hospital care to a patient’s home environment or to appropriate outpatient or inpatient follow-up services [1]. Its primary aim is to ensure continuity of medical and nursing care, prevent complications, and enhance patients’ quality of life. DM can be implemented as a standalone intervention [2], include post-discharge support [3], [4], or be embedded within broader care strategies. Tailored interventions have been developed for various medical specialities to address specific needs. As healthcare systems strive for greater efficiency, safety, and patient satisfaction while reducing costs, DM has gained increasing importance. In Germany, discharge planning approaches have been in place since the 1990s [5]. The legal framework for DM, as stipulated in § 39 (1a) of the German Social Code Book V (SGB V), was formally established through a joint agreement between the National Association of Statutory Health Insurance Funds, the National Association of Statutory Health Insurance Physicians, and the German Hospital Federation. This framework came into force on 1 October 2017 and has since undergone several revisions [1]. It aims to ensure seamless care transitions through close collaboration between hospital and community-based services, as well as through the involvement of family members and care providers. 

Rural areas face particular challenges in implementing effective and needs-oriented discharge processes, due to limited healthcare infrastructure and longer travel distances to specialised services. (The Federal Institute for Research on Building, Urban Affairs and Spatial Development (BBSR) defines rural areas as regions with a population density of fewer than 150 inhabitants per square kilometre, where more than two-thirds of the population live in towns with fewer than 20,000 residents or in rural settlements.) The federal state of Mecklenburg-Western Pomerania (MV), with only 70 inhabitants per square kilometre, has the lowest population density in Germany [6]. Since 1990, its population has declined by 15% [7], while the proportion of residents over the age of 65 has doubled [8], placing additional strain on essential public services. Designing an effective DM approach is therefore of particular relevance for MV. However, research on implementation strategies and stakeholder preferences in rural settings remains limited.

The collaborative research project NAHVERSORGT, conducted by the Universities of Applied Sciences Stralsund and Neubrandenburg, addresses these challenges by evaluating current DM practices in MV and deriving systematic recommendations applicable to other rural and structurally weak regions. The present study focuses on the following research questions:

1. In what ways is DM currently organised and implemented within rural regions of MV?
2. What specific needs, challenges, and resources do healthcare professionals perceive in relation to the delivery of DM in rural contexts?
3. Which strategies and practices within DM have demonstrated effectiveness, and under what conditions?

## 2 Methods

A qualitative research design was selected to explore the subjective experiences of healthcare professionals involved in DM and participating in regional round table discussions (RTs). The aim was to generate context-specific insights and formulate practical recommendations for improving DM in rural settings. The study followed a participatory research approach, actively involving key stakeholders responsible for DM in MV. Group discussions (GDs) were chosen as the primary data collection method, as they allow for the gathering and comparison of diverse perspectives [9] and support the co-construction of potential solutions to complex challenges [10]. A semi-structured discussion guide was developed to structure the group sessions. It was informed by findings from a prior scoping review (Petereit et al., under review) and organised into five thematic dimensions (see Table 1 (Tab. 1)). Each dimension included open-ended questions and prompts to facilitate deeper inquiry and ensure consistency across sessions. The guide was pretested and subsequently revised to enhance clarity and usability. Since 2019, RTs have been established in four rural municipalities in MV, each with fewer than 10,000 residents. These sites were selected in coordination with the “Healthy Ageing” strategy group of the MV Health Economy Advisory Board. Each RT comprises approximately 15 voluntarily participating stakeholders from various professional backgrounds, working collaboratively to develop cross-sectoral solutions for improving local healthcare delivery. Challenges related to DM had been identified as a key issue early in the formation of the RTs. Data collection took place between June and July 2024 within these existing regional networks. Participants were fully informed in advance about the study’s objectives, data handling, pseudonymity measures, and their right to withdraw at any time without providing a reason. Participation in the GD was entirely voluntary.

### Data analysis

All data were pseudonymised and subsequently processed and analysed using a category-based approach. The analysis followed the principles of qualitative content analysis with a structured, thematic orientation, as described by Kuckartz [11], allowing for a combined deductive–inductive procedure. Data analysis was conducted using MAXQDA software (Release 24.2.0). Two researchers independently coded the data and collaboratively reconciled the coding framework and category system through consensual coding to ensure reliability and intersubjective agreement.

## 3 Results

During the data collection period, GDs were conducted at three RTs in MV – specifically in Demmin, Pasewalk, and Ueckermünde. The average duration of each session was 71.35 minutes. A total of 30 participants took part in the study (see Table 2 (Tab. 2)), and all contributions were included in the analysis. The data analysis resulted in the identification of eight main categories (MC) comprising a total of 56 subcategories (see Table 3 (Tab. 3)). The following section presents MC2 to MC7, as these reflect the central thematic areas of the study (see Table 3 (Tab. 3)).

### MC2: Collaboration in DM

Within the framework of DM, participants reported cooperation with a range of professional groups as well as non-professional actors, such as neighbourhood volunteers and local associations (e.g. rural women’s organisations). The intensity and structure of collaboration varied depending on the participants’ professional context, but the overarching aim was to ensure and improve access to healthcare services. Collaboration also served as a key strategy for participants to navigate restricted individual authority and structural rigidity:

*“We currently have waiting lists of up to a year. But if I have an urgent case that needs a care home placement, I have to admit the person to hospital so that the hospital social services can organise a place more quickly – which is simply not possible through my regular practice.”* (H105, Pos. 4)

Satisfaction with interprofessional collaboration differed across professions. For example, nurses reported positive working relationships with local palliative care services (SAPV) and the regional care network “HaffNet”, but noted less satisfactory cooperation with rehabilitation clinics.

### MC3: Needs in DM

In rural regions, patients receiving palliative care, individuals with neurological conditions, people living with dementia, and older adults face an elevated risk of inadequate care due to limited access to healthcare services. These groups are already considered vulnerable, and this vulnerability is further exacerbated by structural deficits in rural healthcare provision. There is a particularly high demand for therapeutic services:

*“Palliative patients discharged from hospital – they simply don’t have access to mobile therapists. Which means they stand no chance of receiving something like lymphatic drainage during their final weeks, because there are so few physiotherapists.”* (S103, Pos. 62)

Availability of rehabilitation services is limited. Existing rehabilitation clinics are frequently overburdened or located far from patients’ homes:

*“The problem is the early neurological rehab clinics. A patient has a stroke, receives good acute care in hospital, and then the only option we have is to transfer them to a nursing home.” *(H107, Pos. 72)

Due to a shortage of qualified personnel in dementia care settings, individuals are frequently relocated to facilities distant from their familiar surroundings:

*“We’re now even sending people to care homes in Poland, because the staffing ratios are completely different there. […] They can’t stay in their local community.”* (H107, Pos. 70)

There is a clear need for accessible counselling centres that offer guidance on assistive devices and care options, as well as for a barrier-free infrastructure, particularly in terms of public transport and age-appropriate housing.

### MC4 and 6: Resources and best practices

In rural healthcare contexts, all actors involved such as healthcare professionals and informal caregivers are regarded as valuable social and personal resources contributing to what can be described as a caring community. The strength and structure of such community support varies across regions. Additional resources include innovative service models such as home visits by medical and dental practitioners to care facilities, as well as individual counselling provided by professionals specialised in dementia care or by trained senior advisors. Well established local care networks are seen as essential to effective healthcare delivery in rural settings and are widely considered examples of best practice in the field of DM:

*“I think the network has developed really well. Communication between general practices, outpatient nursing services, and residential facilities works smoothly. […] I believe that is one of the advantages of rural areas—you know each other, and that exchange functions well.”* (S105, Pos. 67)

Another successful example mentioned was the HaffNet network, a regional association of physicians that supports cooperation among medical professionals and facilitates more coordinated and comprehensive patient care.

Although financial resources were only rarely mentioned in the GDs, material resources in rural regions include an increasing availability of accessible and age-appropriate housing. In addition, integrated urban development strategies are seen as a key factor for sustainable planning, as they combine elements such as housing, mobility, environmental concerns, and social infrastructure within a single coherent approach.

### MC5: Problems

Key challenges identified include securing access to day care services, the shortage of short-term care places close to home, and limited availability of long-term residential care and rehabilitation clinics. These shortages often lead to patients remaining in hospital longer than medically necessary or being transferred to follow-up care facilities far from their home region:

*“Right now, we have patients who have been in hospital for three quarters of a year because we simply cannot find a solution.”* (H107, Pos. 70)

There is also a significant lack of general practitioners and specialists, as well as ongoing therapeutic support, such as physiotherapy and occupational therapy:

*“We also have major problems there. In our area, we barely have any occupational therapists. The waiting lists are extremely long.”* (S105, Pos. 58)

Moreover, the ongoing digitalisation process, legal requirements, and bureaucratic barriers have further complicated DM processes. A specific financial challenge arises from competition between neighbourhood helpers and outpatient care services for the €125 monthly relief contribution: *“Often, the care service already claims the €125. And that’s the problem.”* (T106, Pos. 143). Further issues relate to sustainability and the utilisation of support services, which are hindered not only by funding problems but also by a lack of public awareness and transparency: *“This community transport service is a great idea, but many people have no idea how to use it.”* (T106, Pos. 244). In contrast, in some areas demand for age-friendly housing exceeds supply:* “We built 36 new apartments […] and received 120 applications, which we simply couldn’t meet.”* (H106, Pos. 30). Such housing shortages cannot be addressed quickly due to lengthy funding approval processes, limited availability of skilled trades, and organisational limitations on the part of those affected. Modifications to older buildings are often only partially feasible. Finally, social factors play a substantial role in shaping access to care and effective DM. As relatives often live at a distance, their ability to provide support is significantly reduced.

### MC7: Solutions and recommendations

One key approach identified for addressing the complexity of rural healthcare provision in MV is the mapping and consolidation of existing services, as this helps to uncover synergies and reduce duplication: *“Pooling structures and analysing what’s already in place, making that visible […]”* (S111, Pos. 107). Participants saw high potential in mobile information services, such as a travelling information bus, for reaching out to rural populations: 

*“To really reach the rural communities, an info bus wouldn’t be a bad idea […] that would stop in villages at set times, say twice a week.”* (T106, Pos. 133)

There was also a strong call for early counselling and education on topics such as advance directives, power of attorney, care levels, and accessible housing adaptations. The discussions revealed a significant need for mobile healthcare services, including home visits by doctors and allied health professionals. Specialised roles in nursing—such as community health nurses, ErwiN nurses, or VERAHs—were highlighted as potential solutions to bridge gaps in service provision and improve interface coordination. Finally, digital healthcare models such as telemedicine were considered a promising way to improve access to care and enable greater participation in healthcare services for people in remote areas.

## 4 Discussion

The following section discusses the study’s findings in light of the initial research questions:

### 1. How is DM implemented in rural regions of MV?

The implementation of DM in rural regions of MV is significantly constrained by structural barriers. There is a lack of nearby follow-up care services, including rehabilitation clinics, short term care placements, and long-term residential care facilities, as well as an overall shortage of medical specialists and therapists. These deficiencies particularly disadvantage vulnerable groups. Comparable challenges have been documented in the United States, where an urban rural divide restricts access to healthcare due to staffing shortages, inadequate infrastructure, and limited digital services [12]. The findings of the present study confirm that access to continuing care is hindered by systemic shortcomings. However, some rural areas have managed to alleviate these deficits through informal support structures and locally embedded care networks. Strengthening regional coordination models could help address these gaps, enabling the transfer of successful practices to less developed regions.

### 2. What needs and resources do healthcare professionals identify in relation to DM in rural regions?

Healthcare professionals in rural MV highlight the need for better coordinated and more accessible healthcare services. They describe persistent personnel shortages and structural bottlenecks as major obstacles to care provision. Hansen et al. [13] reinforce this perspective, noting that workforce scarcity and excessive workload are key factors limiting rural healthcare delivery. Pohontsch et al. [14] emphasise the important role of general practitioners in rural areas. These professionals often see themselves not only as providers of medical care, but also as long-term companions to their patients. This close and continuous relationship may be a valuable resource in DM, enhancing patient engagement and supporting the continuity of follow-up care. To address workforce challenges, new financing schemes and demand-based care structures are required, such as locally available short-term care and specialised rehabilitation services. Redundant systems should be avoided and existing resources used more efficiently. Community-based support models can provide targeted supplements. Digital healthcare innovations and telemedicine hold potential to improve service access, though this is hindered by limited internet infrastructure, which remains a challenge in many parts of rural MV [15].

### 3. Which measures have proven effective in DM?

The findings suggest that improved access to specialised services, such as local therapy options or community transport, combined with stronger coordination among healthcare professionals, are particularly effective in supporting DM. Hengel et al. [16] highlight how regional inequalities in healthcare largely concern access and quality—factors that directly affect the effectiveness of DM. Well-functioning local networks can optimise the discharge process by fostering closer collaboration. In rural regions with few follow-up services, this requires robust coordination between hospitals, general practitioners, and community-based care providers.

### Limitations and implications

The study was conducted in accordance with the quality criteria for qualitative research by Steinke [17], [18]. To ensure transparency and reproducibility, all steps of data analysis were documented using MAXQDA software. The coding was conducted by two researchers using a standardised procedure and a combination of deductive and inductive reasoning. Empirical grounding [18] was ensured through the coding structure, the link to a prior scoping review (Petereit et al., under review), and the systematic derivation of findings from the data. The study demonstrates internal coherence and is of practical relevance [18], as it provides insight into the implementation of DM in rural regions. 

Nonetheless, several limitations must be considered. The sample consisted solely of participants involved in RTs in MV. It cannot be ruled out that additional group discussions with healthcare professionals in other regions might have produced different findings or emphases. The voluntary nature of participation may also have introduced selection bias, as more motivated and engaged individuals were likely overrepresented. These participants may already play an active role in shaping regional DM. This limits the transferability of the findings to other regions or to DM across Germany. Although four RTs were originally planned, one had to be excluded due to organisational issues. In the three remaining RTs, participation on the data collection days was lower than anticipated, meaning that not all planned stakeholder groups were represented. The discussion guide was developed by the project team and reviewed by an expert advisory board to minimise bias. However, the phrasing of questions, body language, or reactions from the discussion facilitator may still have influenced participant responses [19].

The findings from this study provide the foundation for further research within the broader research consortium, including a quantitative hospital survey in MV and additional qualitative studies. The overarching aim is to deepen understanding and develop practice-oriented recommendations for DM in rural MV.

## 5 Conclusion

The findings of this study indicate that DM in the rural regions of MV faces specific structural challenges. There is a clear need for local follow-up services such as rehabilitation clinics, short term care facilities, and sufficient specialised staff to ensure continuous and timely care for patients. The regional deficits described in the GDs disproportionately affect vulnerable patient groups, including people living with dementia, individuals with neurological conditions, and older adults. To address these gaps, care models involving mobile services and flexible telemedicine solutions are essential. These approaches have the potential to improve access to healthcare and make more efficient use of existing resources. Furthermore, they support cross sector collaboration and strengthen regional coordination structures, as demonstrated by the positive experiences with the RTs in MV. In the long term, it is recommended that funding opportunities for innovative care models be explored in order to support integrated and sustainable healthcare provision in rural areas.

## Abbreviations

- DM=discharge management
- GD=group discussion
- MV=Mecklenburg-Western Pomerania
- MC=main category
- RT=round table

## Notes

### Acknowledgements

We would like to express our sincere thanks to the members of the round tables for their participation in the voluntary group discussions.

### Funding

This work is part of the collaborative research project “NAHVERSORGT – Regionally Integrated Care”. The project underlying this publication was funded by the Innovation Fund of the Federal Joint Committee (Gemeinsamer Bundesausschuss) under funding code 01VSF23038.

### Ethics statement

Ethical approval for the study was granted by the Ethics Committee of Neubrandenburg University of Applied Sciences (Ref. No.: HSNB/216/24).

### Competing interests

The authors declare that they have no competing interests.

## Figures and Tables

**Table 1:**
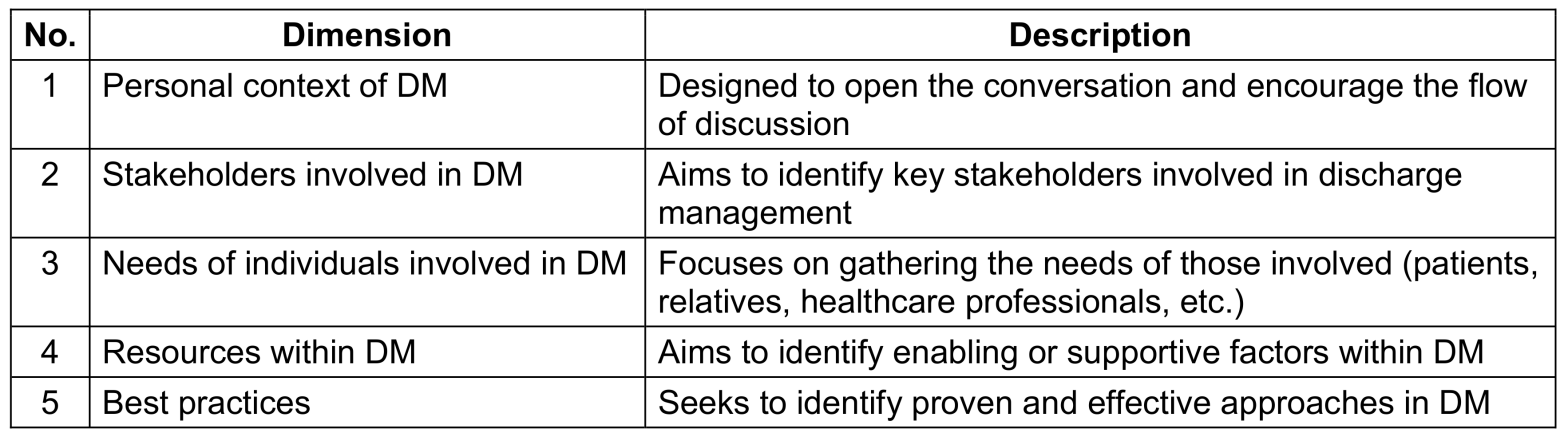
Dimensions of the discussion guide

**Table 2:**
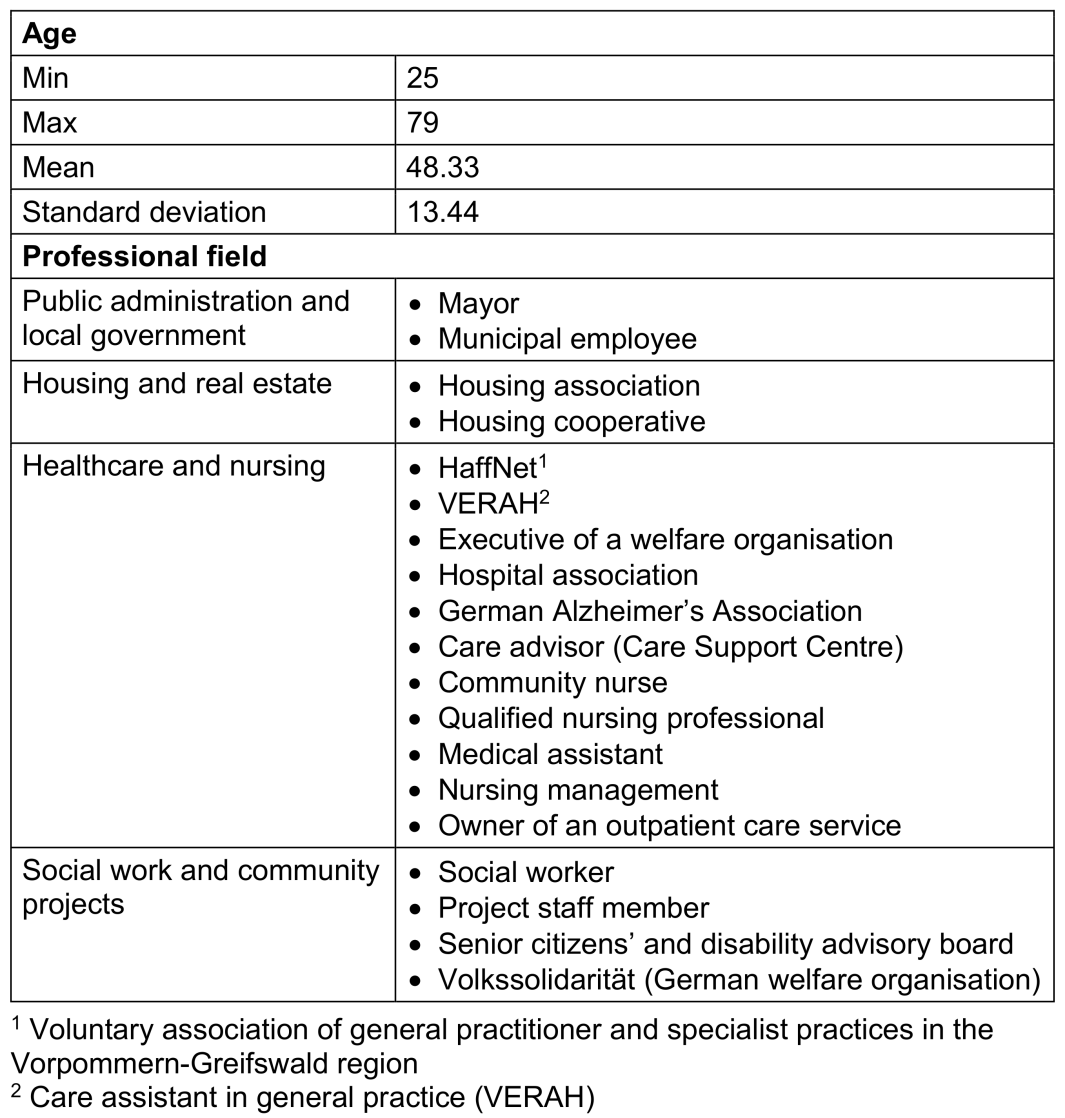
Sample

**Table 3:**
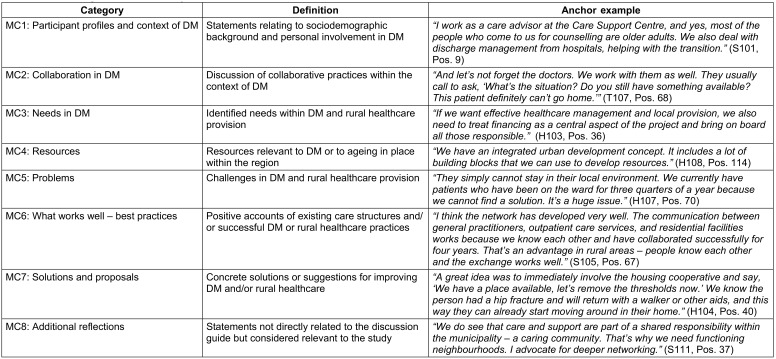
Main categories of data analysis
